# International Glossina Genome Initiative 2004–2014: A Driver for Post-Genomic Era Research on the African Continent

**DOI:** 10.1371/journal.pntd.0003024

**Published:** 2014-08-21

**Authors:** Alan Christoffels, Dan Masiga, Matthew Berriman, Mike Lehane, Yeya Touré, Serap Aksoy

**Affiliations:** 1 South African Medical Research Council Bioinformatics Unit, South African National Bioinformatics Institute, University of the Western Cape, Bellville, South Africa; 2 Molecular Biology and Bioinformatics Unit, International Centre of Insect Physiology and Ecology (ICIPE), Nairobi, Kenya; 3 The Wellcome Trust Sanger Institute, Wellcome Trust Genome Campus, Hinxton, United Kingdom; 4 Vector Group, Liverpool School of Tropical Medicine, Liverpool, United Kingdom; 5 Vector, Environment and Society Unit, Tropical Diseases Research (TDR), World Health Organization, Geneva, Switzerland; 6 Yale School of Public Health, Department of Epidemiology and Public Health, New Haven, Connecticut, United States of America; National Institute of Allergy and Infectious Diseases, United States of America

Human African trypanosomiasis (HAT), also known as sleeping sickness, is a neglected disease that impacts 70 million people distributed over 1.55 million km^2^ in sub-Saharan Africa and includes at least 50% of the population of the Democratic Republic of the Congo [1]. *Trypanosoma brucei gambiense* accounts for more than 98% of the infections in central and West Africa, the remaining infections being from *Trypanosoma brucei rhodesiense* in East Africa [2]. The parasites are transmitted to the hosts through the bite of an infected tsetse fly. Disease control is challenging as there are no vaccines, and effective, easily delivered drugs are still lacking. Treatment invariably involves lengthy hospitalization, with both medical and socioeconomic consequences. Control of disease can be accomplished, however, through vector control, which largely to date has aimed to reduce insect populations rather than eliminate them. In the mid-1990s, disease cases were increasing but tsetse research and facilities that could maintain tsetse fly colonies were on a decline globally, particularly in Africa. This was also at a time when new scientific advances were being realized in other vector-borne disease systems, particularly building on the revolution in genome sequencing and genome-wide analyses.

In 2004, the Molecular Entomology program of the World Health Organization Tropical Diseases Research (WHO-TDR) unit began an initiative (International *Glossina* Genome Initiative, IGGI) that brought together an interdisciplinary group of researchers from multiple countries and institutions to explore possibilities for genomics and post-genomics research in the Tsetse field. The first major goal of IGGI was to produce a reference genome sequence for single Glossina species—work that is described in [3]. However, alongside this direct output, there were two further goals for this initiative. First, to understand the genomic basis of unique aspects of tsetse biology with a focus on applied discoveries, those that could be exploited to aid in vector control; and second, to use this network to help build global capacity, in Africa and globally, for genetics and genomics-based research into tsetse. This vision was crystalized at the outset with a decision to generate sustainable genomic resources on the African continent, and in turn to use these resources to provide training in genome analytical skills (Figure 1). The IGGI consortium met annually to organize training workshops, to initiate transcriptomics-based projects, to recruit financial resources to undertake the WGS initiative and to track progress once the project was successfully launched. Wellcome Trust funding for the WGS project provided the much-needed momentum to ensure the project remained on track.

**Figure 1:**
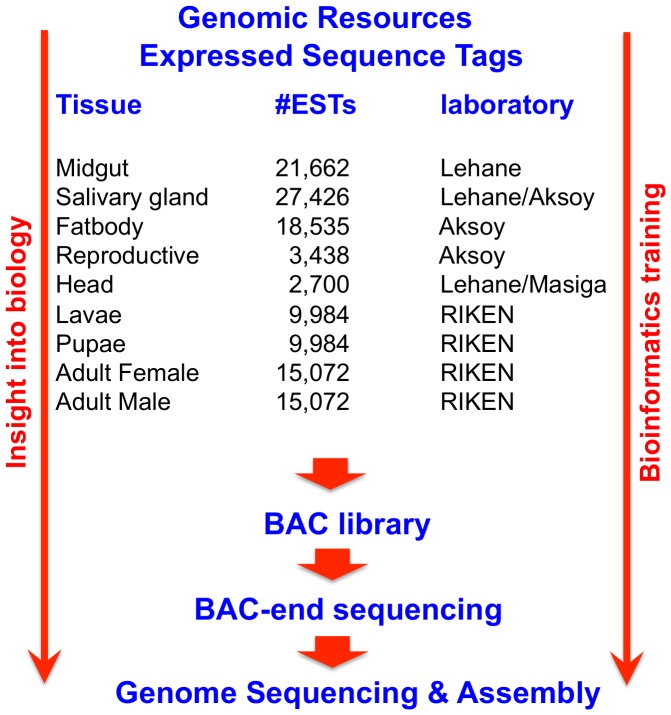
Glossina molecular toolbox to facilitate genomics training. The international Glossina Genome Initiative developed genomic resources including ESTs, a BAC library, and the sequenced *Glossina morsitans mortisans* genome to facilitate genomic capacity development on the African continent.

## Data Curation as a Model for Genomics Training

In lieu of a completely sequenced *Glossina* genome, the consortium recruited global funds that enabled the development of a molecular toolbox, which initially included the production of expressed sequence tags (ESTs)—sequences produced from the ends of cloned cDNA fragments—from 11 large cDNA libraries, along with the construction and sequencing of a bacterial artificial chromosome (BAC) library. The molecular toolbox became the training instrument for five ten-day bioinformatics workshops held at the University of the Western Cape, South Africa, involving a total of 75 students and researchers from 18 African countries (Table 1). Although, by their very nature, the EST libraries only offered a partial and highly fragmented picture of the genome, they were nonetheless an opportunity to gain insight into the molecular mechanisms and processes that govern vectorial capacity of *Glossina* [4]–[9]. These workshops provided an environment for students to interact with international experts from Yale University, European Bioinformatics Institute, Wellcome Trust Sanger Institute, Liverpool School of Tropical Medicine, and RIKEN. The manual annotation exercise of the *Glossina* transcriptome resulted in a curated *Glossina* dataset (www.vectorbase.org) and afforded graduate students an opportunity to acquire a skill set that could be transferred to other sequencing projects. At least 20% of participants have subsequently forged international collaborations with these trainers or have, themselves, become sought after as genomics trainers and supervisors of graduate students.

**Table 1:** Number of participants trained through the International *Glossina* Genome Initiative.

	Bioinformatics Workshops					Transcriptome Jamborees	International Exchange Program (two-months)
Country	2002**	2003**	2004	2006	2008	2007/2008:	2010/2011
Burkina Faso	1	0	0	0	0	1	0
Cameroon	0	0	1	1	1	0	0
Congo	0	0	0	0	1	0	0
Ethiopia	1	0	0	0	1	0	0
Gabon	0	1	1	0	0	0	0
Ghana	0	1	0	0	1	0	0
Ivory Coast	0	0	0	0	2	0	0
Kenya	2	1	3	4	3	11	1
Malawi	0	0	0	1	0	0	0***
Mali	1	1	1	0	1	0	0
Nigeria	2	2	3	1	2	1	0
South Africa	4	2	0	1	0	13	1
Sudan	0	2	2	0	1	0	0***
Tanzania	0	1	1	0	0	1	1
The Gambia	0	0	1	0	0	0	0
Tunisia	2	2	1	0	0	2	0
Uganda	1	0	0	3	2	3	0***
Zambia	1	0	0	0	0	0	0***
Japan*	0	0	0	0	0	4	0
UK*	0	0	0	0	0	13	0
US*	0	1	0	1	0	3	0
							0

* International trainers participating in the workshops.

** These courses were funded before the IGGI consortium was established.

*** Sites identified for future exchange programs.

## International Student Exchange Programs

The IGGI consortium held annual executive meetings, which either coincided with workshop activities or at scientific meetings where many members were present. During the annual IGGI consortium meeting in November 2009, the tsetse biological laboratories from within the consortium established themselves as remote mentoring sites for the *Glossina* Functional Genomics Network. This enabled three African students to participate in a two-month exchange program hosted annually in laboratories in the United States and Europe. These graduate students gained additional skills in genomics and functional genomics areas, and the visits provided them with an established scientific network for academic support while they continued their graduate studies. The first cohort of the exchange program was subsequently funded to present their work at the bi-annual African Society for Bioinformatics and Computational Biology meeting in Cape Town, South Africa in 2011. These students have afterwards either completed their PhD degrees or are in their final stages of doing so. Four additional centers on the African continent were identified as partners within the *Glossina* Functional Genomics Network—in Malawi, Zambia, Sudan, and Uganda.

The skills development funding obtained from the WHO-TDR program was maximized to create a research-enabling environment in tsetse-endemic countries by supporting recipients of the exchange programs. To this end, in collaboration with Professor Erik Bongcam-Rudloff, Uppsala University, the EBIOKIT was adopted for African tsetse research laboratories to carry out genomic analyses locally without the need to rely on internet connectivity. The bioinformatics computing environment was rolled out in Kenya, where 44 participants received hands-on training to use the myriad of bioinformatics analytical tools embedded within the EBIOKIT. This international collaborative training session impacted Egerton University's Faculty of Agriculture, Jomo Kenyatta University of Science and Technology, Moi University, Kenya Wildlife Services, and the Kenyan Ministry of Higher Education.

## African Institution Partnerships

The impact of the IGGI consortium activities over the past eight years can be measured by the capacity development activities afforded to both students and junior researchers in Africa. The IGGI activities have been a catalyst for graduate training in genomics on the African continent. Notwithstanding the training workshops, at least ten MSc and 12 PhD students were trained as a direct consequence of access to Glossina genome data (Table S1). Having an established network allowed some of the African participants to gather additional research funds from the WHO-TDR Entomology Committee to begin a population genetics investigation in the Lake Victoria Basin. This initiative further enabled the network to recruit three PhD students to be trained in genomics research. Similarly, the Yale scientists within IGGI were successful in securing funds from the Fogarty International Center to help support capacity in tsetse research in East Africa. These funds allowed the network participants to organize workshop activities in Kenya and Uganda focusing on population genetics/genomics, bioinformatics, and functional genomics fields for tsetse researchers and students.

Researchers, on the other hand, who have been part of the IGGI consortium, have facilitated short courses at their local institutions; been invited to act as reviewers for international journals; and won competitive grants from IAEA, WHO-TDR, BecA/NEPAD, National Institutes of Health (NIH), Fogarty International Center of NIH, and the South African National Research Foundation. More recently, networks have been established to enable graduate students to be cosupervised across African institutions where experimental work is being carried out at International Centre of Insect Physiology and Ecology (ICIPE) and the Trypanosomiasis Research Center (TRC) in Kenya, Gulu University and Makerere University in Uganda, as well as at Yale University in the US, and the bioinformatics analysis and training is conducted at the South African National Bioinformatics Institute in South Africa.

## Conclusion

The genomic activities of the IGGI consortium over the past ten years have provided a skill set for African researchers to exploit the recently sequenced *Glossina morsitans morsitans* genome. The completion of a ten-year genome project has cemented a scientific network of trypanosomiasis researchers that will be required to provide mentoring to new students and researchers as strategies are sought to best integrate the valuable genomic resources for the Tsetse and the TriTryp genomes. Moreover, the involvement of scientists from disease-endemic countries from the start sets the Tsetse genome project apart from other vector or parasite projects and will help to ensure a greater sense of ownership and long term commitment. Access to the human genome sequence, the trypanosomatid genomes [10]–[13], and trypanosome ‘omics data [14], [15] has provided insight into host–parasite interactions and the identification of new vaccine candidates or chemotherapeutic targets. Targeting tsetse–trypanosome interactions with the goal of identifying genes that modulate trypanosome transmission has entered the realm of feasibility with the completion of the tsetse genome sequencing project.

## Supporting Information

Table S1 Graduate training programs facilitated through access to the *Glossina* genome data.
(DOCX)
