# Supplementary material for: International Glossina Genome Initiative 2004–2014: A Driver for Post-Genomic Era Research on the African Continent
Source: PLoS Negl Trop Dis. 2014 Aug 21;8(8):e3024. doi: 10.1371/journal.pntd.0003024 (PMC4140670; doi:10.1371/journal.pntd.0003024)
Supplement: Table S1 — Graduate training programs facilitated through access to the Glossina genome data. (DOCX) [file pntd.0003024.s001.docx]

Table S1. Graduate training programmes facilitated through access to the Glossina genome data

| **Name** | **Degree** | **Thesis topic** | **Graduation status** |
| --- | --- | --- | --- |
| Sarah Mwangi | MSc | Comparative analysis of insect SERPINS | Graduate 2009, University of the Western Cape |
| Feziwe Mpondo | MSc | A comparative genomics approach towards classifying immunity-related proteins in tsetse fly | Graduate 2009, University of the Western Cape |
| Rosaline Macharia | PhD | Olfactory responsive genes in selected tsetse species in Kenya | Registered for PhD studies in 2013, University of the Western Cape |
| George Obiero | PhD | Annotation and characterization of chemosensory receptors in the genus Glossina | Thesis submission in 2014, University of the Western Cape |
| Sarah Mwangi | PhD | Characterisation of Glossina promoters | Thesis submission in November 2013, University of the Western Cape |
| Oliver Kijanga | PhD | Population dyanamics of *G.fuscipes fuscipes* in the Lake Victoria Basin, Tanzania | Thesis submission in December 2013, |
| Florence Wamwiri | PhD | Tsetse symbionts | Graduate of Unviersity of Egerton, 2013, |
| Richard Echodu | PhD | Tsetse Population Genetics | Graduate of Gulu University, Uganda 2013 |
| Agapitus Kato | PhD | Tsetse Population Genetis | Registered for PhD studies at Makerere University, Uganda 2012 |
| Robert Opiro | PhD | Tsetse Population Genomics | Registered for PhD studies in 2013, Gulu University Uganda |
| Patrick Abila | PhD | Adaptive divergence and natural selection | Registered for PhD studies in 2013, University of Pretoria |
| Zahra Jalali | PhD | Iron metabolism in *Glossina morsitans* | Thesis submitted August 2013, University of the Western Cape |
| Mark Wamalwa | PhD | Genome annotation and curation framework for the analysis of *G.morsitans* ESTs | Graduated 2011, University of the Western Cape |
| Edwin Murungi | PhD | *T.brucei*-human protein-protein interactions: intracellular trafficking towards the flagella pocket | Graduate 2012, University of the Western Cape |
| Paul Mireji | PostDoc | Glossina Olfactory Molecular Genetics | Postdoctoral Fellow at Yale University, USA |
|  |  |  |  |
